# Biopolymer-Based Nanosystems: Potential Novel Carriers for Kidney Drug Delivery

**DOI:** 10.3390/pharmaceutics15082150

**Published:** 2023-08-17

**Authors:** Hao Li, Wenni Dai, Li Xiao, Lin Sun, Liyu He

**Affiliations:** Department of Nephrology, The Second Xiangya Hospital, Central South University, Hunan Key Laboratory of Kidney Disease and Blood Purification, Changsha 410011, China; lihaoxy@csu.edu.cn (H.L.);

**Keywords:** biopolymer, drug delivery systems, kidney, target, nanomedicine

## Abstract

Kidney disease has become a serious public health problem throughout the world, and its treatment and management constitute a huge global economic burden. Currently, the main clinical treatments are not sufficient to cure kidney diseases. During its development, nanotechnology has shown unprecedented potential for application to kidney diseases. However, nanotechnology has disadvantages such as high cost and poor bioavailability. In contrast, biopolymers are not only widely available but also highly bioavailable. Therefore, biopolymer-based nanosystems offer new promising solutions for the treatment of kidney diseases. This paper reviews the biopolymer-based nanosystems that have been used for renal diseases and describes strategies for the specific, targeted delivery of drugs to the kidney as well as the physicochemical properties of the nanoparticles that affect the targeting success.

## 1. Introduction

Kidney disease is a significant challenge for human health. Kidney disease can be classified into acute kidney injury (AKI) and chronic kidney disease (CKD) [1]. AKI is a pathophysiological condition in which glomerular filtration is acutely reduced over a short period, as evidenced by a rapid increase in serum creatinine, decreased urine output, or both [2,3]. AKI occurs in approximately 10–15% of patients following hospital admission and in intensive care unit (ICU) patients, and it has a 60% chance of causing death [4]. It is a significant public health problem affecting millions of patients worldwide [5]. Moreover, AKI and CKD are closely related; AKI not only promotes the development of CKD, but CKD patients are also at high risk of AKI [6,7]. CKD is defined as a decrease in the glomerular filtration rate or an increase in urinary albumin [8]. The prevalence of CKD among adults aged 18 years or older in mainland China was found to be 8.2% [9]. Currently, the number of people with CKD worldwide is approximately 843.6 million [10]. By 2040, CKD will be the fifth leading cause of loss of life expectancy worldwide [11]. In addition to AKI and CKD, there are several other kidney diseases, including ischemia/reperfusion injury, renal vascular hypertension, and renal tumors. Approximately 5 to 10 million people die from kidney disease every year [12]. Despite the significant health burden of kidney disease, the treatment options are very limited [13]. There is an urgent need for new treatments to manage kidney disease effectively.

Nanotechnology is an emerging technology of the twenty-first century. The application of nanoparticles in treating and diagnosing diseases is an essential area of current nanotechnology research, known as nanomedicine [14]. Nanoparticles are particles with a size of 1–100 nm, including inorganic nanoparticles, lipid nanoparticles, carbon-based nanoparticles, polymer nanoparticles, and biomimetic nanoparticles [15]. There are many methods for the preparation of nanoparticles, which differ based on nanoparticle type. The common preparation methods include desolvation, thin-film hydration, microemulsion, covalent crosslinking, solvent evaporation, and so on [16,17,18]. Nanoparticles used as drug delivery systems can enhance the stability and solubility of drugs, promote drug transport across cell membranes, and prolong their circulation time to boost their safety and effectiveness. Additionally, nanoparticles can help to concentrate drugs at the site of disease or injury, while minimizing their adverse effects [19]. To date, various nanoparticles have been used as carriers for kidney-targeted drug delivery, making important contributions to, and showing great potential for the treatment of renal diseases (Table 1).

Biopolymers are polymers produced by living organisms. Biopolymers have better biocompatibility, bioavailability, and bioreactivity than synthetic polymers [42]. These advantages have led to a wide range of applications in the biomedical field. The combination of nanotechnology and biopolymers in biopolymer-based nanosystems shows great potential for targeted drug delivery [43,44]. The biopolymer-based nanosystems are particularly promising for the treatment of kidney diseases with targeted drug carriers.

This paper focuses on a review of biopolymer-based nanosystems for the treatment of kidney diseases. In addition, kidney-specific targeted delivery strategies are considered, as well as the impact of the physicochemical and functional properties of nanoparticles on the ability to target specific parts of the kidney. The aim of this paper is to identify new approaches for the treatment of renal diseases and to provide some guidance for subsequent studies on kidney-targeted drug delivery.

## 2. Targeted Kidney Strategies

**Figure 1:**
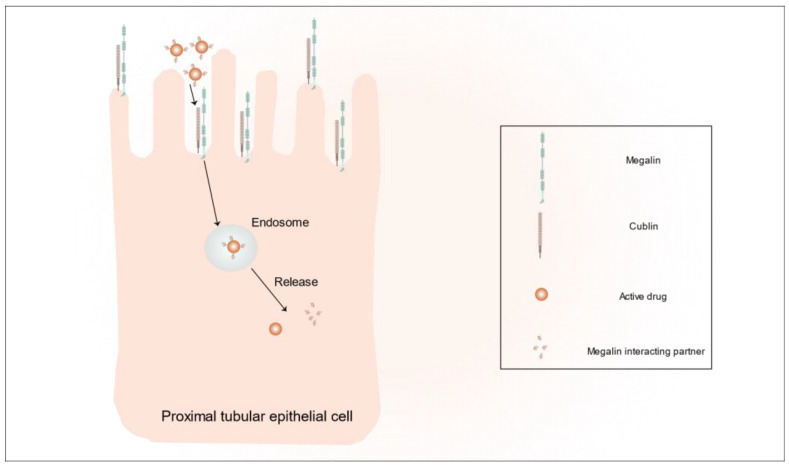
Schematic representation of megalin and cubilin receptor-mediated endocytosis in renal proximal tubular epithelial cells.

Megalin is a receptor protein specifically expressed in human renal proximal tubular epithelial cells and is a member of the LDL receptor family [45]. Megalin is a transmembrane protein that is structurally similar to LDL-associated protein 1, which has an extracellular structural domain of 4600 amino acids with a single transmembrane structural domain and a small cytoplasmic tail [46]. Megalin binds to cubilin at the apical plasma membranes of proximal tubular epithelial cells, and together, they mediate the endocytosis of a large number of different ligands (plasma proteins, peptides, enzymes, vitamin-binding proteins, hormones, and hormone-binding proteins, as well as drugs and toxins) (Figure 1) [47,48]. Due to its differential expression in the human kidney, as compared to other tissues, megalin is currently the dominant target for renal-targeted drug delivery.

Lysozyme, chitosan, and polymyxin B have been shown to bind to megalin receptors on proximal tubular epithelial cells, allowing the drug to be taken up and utilized by the renal tubules through receptor-mediated endocytosis. Lysozyme is a low-molecular-weight protein that is reabsorbed into the proximal tubular epithelial cells after filtration through the glomerulus. Lysozyme has the advantage of being biodegradable and non-immunogenic [49]. Its coupling to the drug gives the drug the ability to selectively target the proximal tubule. The Rho-associated coiled-coil–forming protein kinase (ROCK) inhibitor Y27632 is an effective treatment for renal ischemia-reperfusion injury, but it is also involved in vascular smooth muscle contraction and the migration and activation of immune cells; thus, the systemic use of ROCK inhibitors can lead to adverse effects. After coupling to lysozyme using the Universal Linkage System (ULS), pharmacokinetics confirmed that the Y27632-lysozyme conjugate accumulated rapidly and extensively in the kidney and effectively inhibited ischemia-reperfusion-induced renal tubular injury [50]. Similarly, coupling a TGF-β type I receptor kinase inhibitor (TKI) to lysozyme via a platinum-based ULS ameliorated renal inflammation in vivo among rats via rapid accumulation in the renal tubular cells and inhibited tubular cell and fibroblast activation [51]. In addition, imatinib and sunitinib can also be used to target kidney disease with a platinum-based ULS coupled to lysozyme, allowing for rapid accumulation in renal tubular cells [52,53].

Low-molecular-weight chitosan is a common biopolymer filtered through the glomerulus and reabsorbed via megalin-mediated endocytosis. Low-molecular-weight chitosan can be cleared more rapidly by the kidneys than lysozyme [54]. For example, catecholamine-derived low-molecular-weight chitosan, zinc, and emodin nanoparticles can accumulate specifically in the kidney and effectively treat renal fibrosis in mice [55]. Similarly, chitosan/siRNA nanoparticles can be specifically targeted to proximal tubular epithelial cells in mice because chitosan can specifically distribute and accumulate in megalin-expressing renal tubular epithelial cells [56].

Polymyxin B has been studied and confirmed to have a strong affinity for megalin [57]. Polymyxin B formed positively charged spherical nanoparticles after binding to PEI through an amidation reaction. In vitro studies showed that polymyxin-B-modified nanoparticles could significantly improve the gene transfection efficiency of renal cells. This suggests that polymyxin-B-modified complexes are a drug delivery system with great potential to target the kidney.

In addition to coupling to lysozyme, chitosan, and polymyxin B, the attachment of a renal-targeting peptide to a drug is another strategy for targeted drug delivery to the kidney. Kidney-targeting peptide (KKEEE) _3_K([Lys–Lys–Glu–Glu–Glu]_3_–Lys) can also bind to the megalin receptor in proximal tubular epithelial cells [58,59]. Organic nanoparticles formed after the binding of kidney-targeting peptides to peptide-amphiphilic micelles can cross the glomerular filtration barrier and accumulate in the kidney. By sectioning the kidney sections treated with micelles attached to the kidney-targeting peptide and unattached micelles and performing co-localization analysis via staining for the megalin receptor, it was demonstrated that micelles attached to the kidney-targeting peptide could specifically accumulate in the kidney [35,60]. This novel nanoparticle platform linked to renal-targeting peptides could be used as a candidate drug delivery vehicle to directly treat diseased tissue in kidney disease. In addition, CLPVASC, a phage-display-screened kidney-targeting peptide, can also selectively target the kidney [61]. The addition of a renal-targeting peptide modification to the N-terminus of an elastin-like polypeptide-vascular endothelial growth factor fusion protein slowed the in vivo clearance of the drug and increased renal deposition. The complex was deposited not only in the renal tubules and blood vessels but also in the glomerular foci [62]. Unlike previous renal-targeting compounds, this renal-targeting peptide has the disadvantage that its specific mechanism of uptake by the kidneys is unknown.

Based on the information above, most renal targeting strategies target megalin receptors in the proximal tubule, which is very promising for the targeting of renal tubulointerstitial diseases. For example, diabetic nephropathy usually causes tubular dysfunction and tubulointerstitial fibrosis, and the targeting of the proximal tubule is important for the treatment of diabetic nephropathy [63].

## 3. Influence of the Physicochemical Properties of Nanoparticles on Kidney Targeting

**Figure 2:**
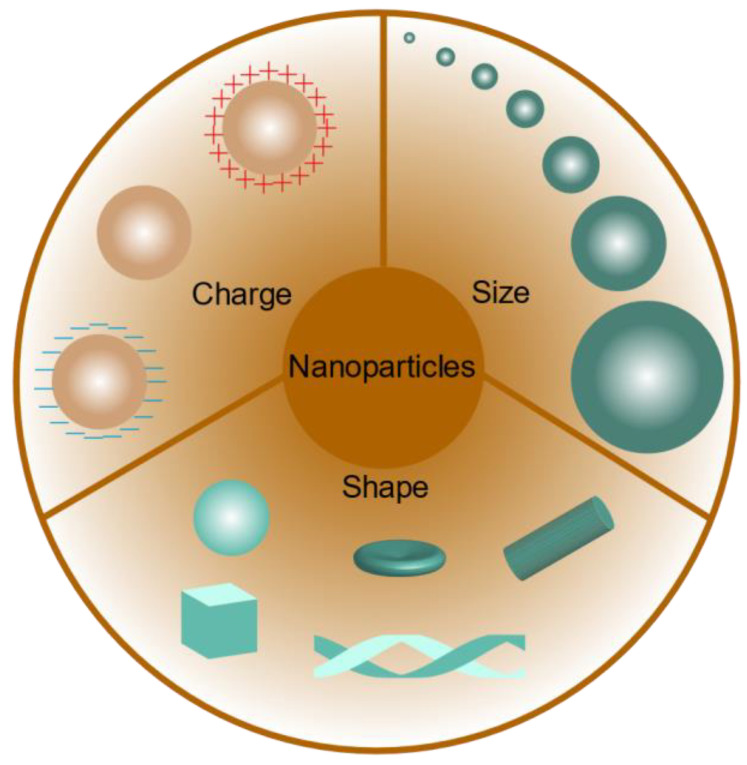
Nanoparticles with different characteristics.

In addition to the methods used to target the kidney with megalin receptors, the physicochemical properties of the nanoparticles themselves affect their targeting ability (Figure 2).

### 3.1. Nanoparticle Size

**Figure 3:**
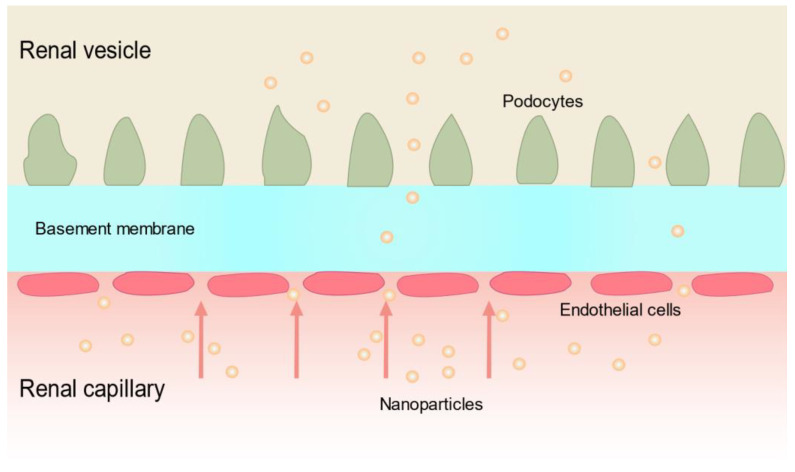
Schematic diagram of the structure of the glomerular filtration barrier.

The particle size of nanoparticles significantly affects their distribution and bioavailability and is one of the key factors affecting their organ targeting [64]. Particles that are too small are quickly excreted from the body by the kidneys, while particles that are too large cannot pass through the kidney’s filtration barrier to reach the inside of the kidney in order to function. Thus, to understand this process, one must consider the filtration barrier of the kidney. For a drug to reach the kidney cells from the bloodstream, it must pass through the glomerular filtration barrier. The glomerular filtration barrier is a highly specialized capillary wall composed of three parts: the endothelial cells, the podocytes (Figure 3), and the basement membrane. Among these components, the glomerular endothelial cells have the function of opening windows, with a pore size of approximately 70–100 nm, while the slits between neighboring podocytes are approximately 32 nm, and the pore size of the basement membrane is approximately 3 nm [65,66]. In healthy kidneys, particles with sizes of 5–7 nm can reach the kidneys through the glomerular filtration barrier, while nanoparticles smaller than 5.5 nm are rapidly cleared by the kidneys [63,67]. However, nanoparticles smaller than 10 nm in diameter are often rapidly excreted from the kidneys, while large particles are deposited primarily in the lungs, liver, or spleen [64]. It has been shown that mesoscale nanoparticles with a particle size of approximately 400 nm can be selectively targeted to the renal tubules, and the localization efficiency in the kidney is 26–94 times higher than that in other organs [68,69]. These nanoparticles have also been successfully used to package drugs for the treatment of AKI-induced renal tubular inflammation, necrosis, and apoptosis [70]. In fact, the tubular filtration barrier can be impaired due to renal diseases, such as diabetic nephropathy, which causes glomerular podocyte detachment and reduced endothelial cell opening [71,72]. Therefore, the vast majority of therapeutic nanoparticles have a particle size of 30–150 nm [73].

### 3.2. Nanoparticle Charge

The glomerular filtration barrier, in addition to its mechanical barrier role mentioned above, also has the role of a charge barrier which influences the ease with which nanoparticles can pass through the glomerular filtration barrier. The glomerular capillary wall is negatively charged, and due to the charge interaction, for nanoparticles with the same particle size, positively charged nanoparticles are more likely to pass the glomerular filtration barrier than negatively charged or neutral nanoparticles [63,74]. In addition to focusing on the filtration barrier of the kidney, we should also focus on the passage rate of nanoparticles in the circulation. In circulation, highly positively charged nanoparticles are rapidly removed from the circulation by mononuclear phagocytes due to increased binding to proteins or high-affinity interactions with mononuclear phagocytes [75]. Nanoparticles with a surface charge <15 mV have minimal macrophage uptake and longer circulation times [76]. Therefore, a surface charge that is positive and less than 15 mV appears to be the best choice of charge for targeted renal drug delivery.

### 3.3. Nanoparticle Shape

The shape of nanoparticles influences their blood rheology, macrophage uptake, cell internalization, and drug-loading capacity [77,78]. Therefore, their shape has significant impacts on the in vivo properties and biodistribution of nanoparticles. With technological advances, nanoparticles have been produced in many diverse shapes, including spherical, cubic, hexagonal, spiral, elliptical, rod, and prismatic forms [1]. Flat, rounded nanoparticles may survive longer in circulation due to their lower uptake by macrophages [79]. In contrast to spherical nanoparticles, non-spherical nanoparticles are more susceptible to tumbling and oscillation effects in the circulation, which greatly increases the likelihood of nanoparticle contact with the cell wall and potential extravasation through open windows in the vasculature [77]. There are little data available on the study of different shapes of nanoparticles for kidney-targeted drug delivery, but from the numerous studies of nanocarriers for this purpose, the spherical shape seems to be the strategy in most cases [63].

## 4. Biopolymers and Their Application in Renal Diseases

### 4.1. Chitosan

**Figure 4:**
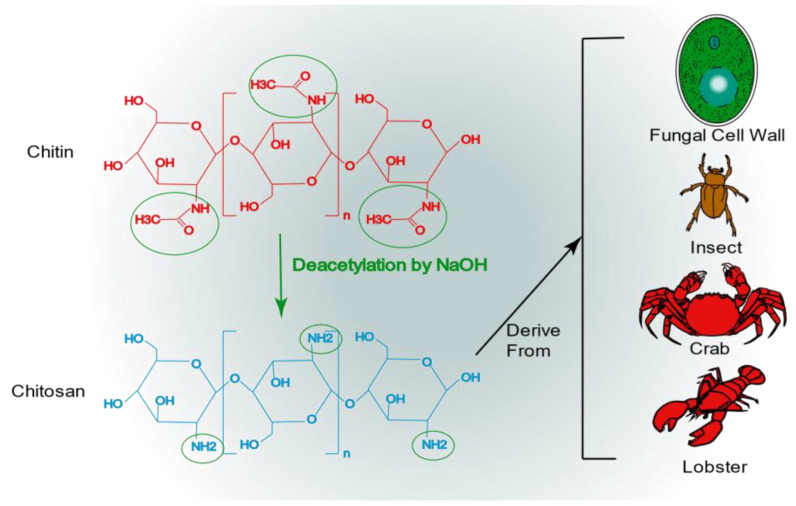
The chemical structure of chitosan and its origin.

Chitosan is a highly abundant natural biopolymer derived from the exoskeletons of crustaceans such as lobsters and crabs, insects, and fungal cell walls [80]. Chitosan is a polysaccharide formed through the deacetylation of chitin, and its chemical structure consists of N-acetylglucosamine and D-glucosamine monomers (Figure 4). Chitosan’s molecular formula is C_6_H_11_NO_4_X_2_. Chitosan is formed after the N-deacetylation of chitin in reaction with a concentrated NaOH solution [81]. After deacetylation, as compared to chitin, chitosan acquires a higher water solubility and better chemical potential due to the free amino group [82]. The amino group on the surface of chitosan results in its positive charge [42]. This is of great interest for drug delivery to the kidney, as positively charged substances are more easily transported across the glomerular filtration barrier [63]. With continuous technological progress, the third generation of chitosan was introduced in the form of chitosan oligosaccharides, and chitosan became a designable bioactive compound, which greatly enhanced its application prospects [83]. At present, chitosan has a wide range of applications, including not only antibacterial, antitumor, and antioxidant applications [84,85,86] but also drug delivery, especially in its nanoform [87,88,89]. Chitosan nanoparticles are easy to produce, have a low toxicity, and are highly stable, biocompatible, and biodegradable. In addition, chitosan nanoparticles can release their encapsulated chemicals in a controlled manner [90]. As previously mentioned, chitosan with a low molecular weight can be selectively absorbed by megalin receptors in the kidney. Consequently, both chitosan nanoparticles and hybridized nanoparticles containing chitosan are effectively and selectively distributed in the kidney. All these advantages make chitosan nanoparticles an excellent vehicle for drug delivery to the kidney.

As stated in the previous section, chitosan nanoparticles are excellent carriers for drug delivery to the kidney. C. Prajapati et al. [91] synthesized N-acetylated chitosan nanoparticles capable of renal targeting for the encapsulation of thymoquinone (TQ) to treat hemorrhagic cystitis. They successfully prepared a TQ-nanoparticles formulation using an ionic gelation technique with N-acetylated chitosan, tripolyphosphate, TQ, and methanol. The structure of TQ-nanoparticles is spherical, with an average particle size of 272.6 nm, a polymer dispersity index of 0.216, and a zeta potential of −20.7 mV. TQ-nanoparticles have good bioavailability, water solubility, and renal targeting. The renal targeting of TQ-nanoparticles is effected through the specific uptake of N-acetylated chitosan by the megalin receptors of the renal tubular epithelial cells [92]. After uptake, the drug achieves maximum accumulation in the kidney, leading to a massive release of the drug into the bladder, which results in the treatment of hemorrhagic cystitis.

Chitosan nanoparticles have important applications not only in hemorrhagic cystitis but also in the removal of harmful reactive oxygen species (ROS) from the kidney. M. Ahmad et al. [93] synthesized chitosan and curcumin composite nanoparticles through an ionic crosslinking method using glutaraldehyde as the crosslinking agent. They demonstrated that chitosan nanoparticles can have a strong effect as a biosorbent for the removal of ions such as cadmium or copper from the solution. The authors prepared nanoparticles with particle sizes in the range of 2–40 nm, with good physical and chemical stability and easy dispersion in water. Curcumin, a well-known natural antioxidant, has a good ameliorating effect on oxidative stress induced by the toxic metal cadmium in the kidney when combined with chitosan nanoparticles. Similarly, M. Pang et al. [94] used low-molecular-weight chitosan to encapsulate lutein and celastrol in order to prepare nanomicelles that could be delivered to the kidney for the treatment of acute kidney injury (AKI). Nano micelles measuring 75.0 ± 5.0 nm in size were created with a consistent spherical shape, a zeta potential of 20.0 ± 3.2 mV, and a polymer dispersity index (PDI) of 0.13 ± 0.05. They cleverly linked citraconic anhydride to low-molecular-weight chitosan through amide bonding, linked the lutein to citraconic anhydride through an esterification reaction, and finally added the lipid-soluble compound celastrol to the hydrophobic core of the micelles. Moreover, due to the presence of amide bonds in these nanomicelles, which can be broken under acidic conditions, these nanomicelles also possess PH sensitivity, which contributes to the rapid release of the drug in the acidic environment of lysosomes after uptake by the cells. The renal targeting of these nanomicelles is due to the fact that low-molecular-weight chitosan can be specifically taken up by megalin receptors in renal tubular epithelial cells, similar to the mechanism for the specific uptake of TQ-nanoparticles by the kidney, as mentioned previously [95]. The average particle size of the nanomicelles was 75.0 ± 5.0 nm, with a uniform spherical shape, PDI of 0.27 ± 0.04, and zeta potential of 20.0 ± 3.2 mV. The distribution of the fluorescently labeled nanomicelles in mice was observed via NIR imaging, and it was demonstrated that the nanomicelle group had a stronger renal fluorescence intensity compared to the control group and showed rapid accumulation in the kidney.

Nanozymes are a focal topic of current research. Nanozymes are nanomaterials with an enzyme-like activity that have effective catalytic activity and a specific mechanism for a given reaction [96]. Z. Liu et al. [97] designed an ultra-small nanoenzyme for the treatment of AKI, and they successfully synthesized the nanoenzyme with an average particle size of 2 nm using chitosan, ruthenium(III) chloride trihydrate, and acetic acid with a solvothermal method. In addition, the nanoenzyme possesses multienzyme-like activity (superoxide dismutase (SOD), catalase (CAT), and glutathione peroxidase (GPx)) and acts as an antioxidant to effectively scavenge ROS in kidney cells for the treatment of AKI. The excellent antioxidant properties, biostability, biocompatibility, and renal accumulation of this nanoenzyme make it promising for further applications in other kidney diseases caused by ROS, such as diabetic kidney disease.

### 4.2. Cellulose

**Figure 5:**
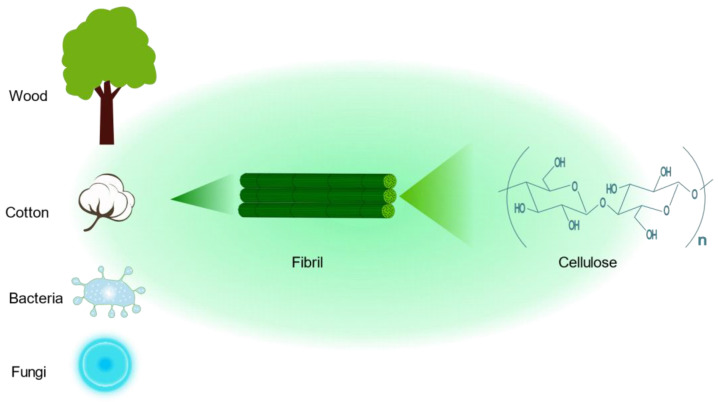
The chemical structure of cellulose and its sources.

Cellulose is by far the most abundant, most common, and renewable natural biopolymer, which is derived from wood, cotton, bacteria, and fungi [98,99]. Cellulose is a polysaccharide with a linear structure consisting of multiple D-glucose units linked by β-1,4 glycosidic bonds (Figure 5) [100]. The cellulose molecular formula is (C_6_H_10_O_5_)_n_. Cellulose has a highly biocompatible and biodegradable nature, making it non-toxic [101]. Although cellulose is insoluble in water, which may limit its application in drug delivery, a series of cellulose derivatives can be formed through specific chemical reactions to improve its water solubility [102]. There are crystalline and amorphous regions in cellulose, and nanocrystalline cellulose is pure cellulose in a crystalline form with nanoscale dimensions [103]. The preparation methods of nanocrystalline cellulose mainly include acid hydrolysis, esterification using concentrated organic acids, (2,2,6,6-tetramethylpiperidin-1-yl)oxidanyl(TEMPO)-mediated oxidation, microbial or enzymatic hydrolysis, and periodate oxidation [104]. Nanocrystalline cellulose is a rod-shaped particle with an excellent aspect ratio, with a width of approximately 5–30 nm and a length of approximately 100–500 nm [105]. It also has superior axial Young’s modulus, high specific surface area, high transparency, and a number of other excellent properties, References [106,107]. As a result, nanocrystalline cellulose is widely used not only in drug delivery but also in many fields, such as papermaking, food production, and electronics [108,109,110]. In the kidney, nanocrystalline cellulose with a high aspect ratio (>10) is aligned with blood flow, filtered through the glomerulus, and subsequently reabsorbed by the renal unit from the tubular border. Due to the pharmacokinetic profile of the delivery platform, drugs bound to nanocrystalline cellulose are delivered to renal tubular cells [111]. Therefore, nanocrystalline cellulose has tremendous potential for renal drug delivery.

Cellulose nanocrystals have a wide range of applications in drug delivery due to their chemical modifiability and biocompatibility, e.g., breast cancer, liver cancer, and myocardial diseases [112,113,114]. Hyperphosphatemia is one of the major metabolic disorders in CKD, and serum phosphate concentration is positively correlated with mortality in patients with advanced CKD [115]. Currently, the treatment of hyperphosphatemia in CKD patients is mainly based on dialysis and oral phosphate binders. Recently, Qimeng Zhang et al. developed cationic cellulose nanocrystals for the treatment of hyperphosphatemia, which can greatly reduce the blood phosphate level [116]. Cationic cellulose nanocrystals have a much larger surface charge than ordinary unmodified cellulose nanocrystals, which enables them to better bind anions (such as phosphate particles). Cationic cellulose nanocrystals were obtained via the wet and semi-dry process incorporation of cationization agents (e.g., glycidyltrimethylammonium chloride) into alkaline-activated cellulose nanocrystals [117]. The authors of the study designed cationic cellulose nanocrystals, which are rigid rod-shaped nanoparticles with a diameter of approximately 10 nm, a length of approximately 150 nm, a large specific surface area, and a zeta potential of 62.3 mv. The cationic cellulose nanocrystals relied on electrostatic attraction and ion-exchange reactions to adsorb phosphate ions, with a binding capacity of approximately 74.3 mg/g, which is significantly higher than that of the commonly used phosphate binding agents (e.g., calcium carbonate, with approximately 39 mg/g) [118]. The authors used these cationic cellulose nanocrystals in mice with chronic renal failure, and the serum phosphorus level of the model mice rapidly decreased to the same level as that of normal mice. The cationic cellulose nanocrystals were able to treat hyperphosphatemia by binding phosphate in the intestinal tract and effectively improved the inflammatory infiltration of the kidneys. In a study conducted by Sam Wong et al., strategic modifications of the primary and secondary hydroxyl groups on cellulose nanocrystals were performed through the introduction of amine and iodine substituents, respectively, were designed to serve as a potential drug delivery platform for the kidney [119]. The cellulose nanocrystals had a rod-like shape with a length of 164.0 ± 20.3 nm, a diameter of 10.52 ± 0.76 nm, and an aspect ratio of 15.97 ± 1.73 and exhibited good biocompatibility and safety, which allowed for their specific accumulation in the kidney, with an accumulation of 23% IA/g within 1 h after intravenous drug injection and the presence of 15% IA/g.

There are a large number of extensive studies on the synthesis, characterization, and biological interactions of cellulose nanocrystals, as well as the successful loading of various hydrophobic drugs. However, there are few studies on the use of these drugs for the treatment of renal diseases [120,121]. For example, curcumin, a polyphenolic compound with antioxidant properties, can be incorporated into cellulose nanocrystals modified with the cationic surfactant cetyltrimethylammonium bromide to bind a large amount of curcumin, and the amount of curcumin added ranges from 80% to 96% [122]. Moreover, curcumin, as an antioxidant, has great application in alleviating oxidative stress, such as diabetic nephropathy and AKI [123,124]. Therefore, cellulose nanocrystals with certain modifications have great potential and innovative applications in the treatment of kidney diseases.

### 4.3. Alginate

**Figure 6:**
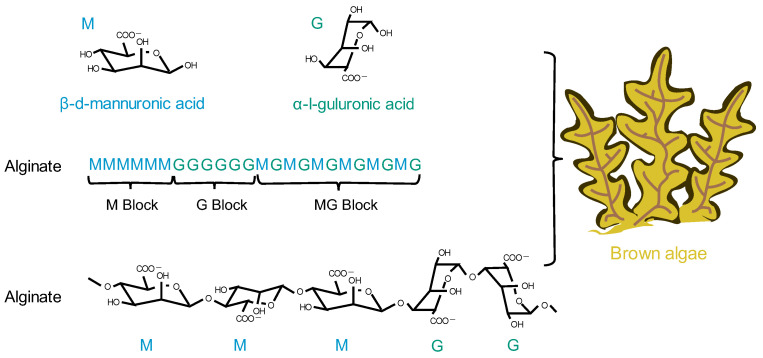
The chemical structural formula of alginate and its origin.

Brown algae are the largest group of marine macroalgae, and alginates are the most abundant natural marine polymers derived from brown algae [125]. Alginate is a non-branched polysaccharide consisting of (1,4) linked β-D-mannuronic acid (M) and α-L-guluronic acid (G) in a non-repeating block. It consists of M residues and G residues that form M blocks or G blocks, respectively, or alternately, both form MG blocks (Figure 6) [126]. Alginate has a wide range of applications, from textile and food technology to biomedical and chemical engineering [127,128,129]. It has important applications in wound dressings; when applied to wounds, it forms a protective gel layer that promotes wound healing and tissue regeneration and maintains a stable temperature. In addition, alginate has applications in drug delivery due to its variable density and fiber composition, which make it easy to control the rate of drug release [130]. In addition, alginate can be easily manipulated through the simple addition of crosslinking agents, such as divalent calcium ions, for the development of different formulations of carriers [131]. Among the various alginate formulations, alginate microspheres have been extensively studied for their ability to encapsulate molecules with different properties. It is worth noting that alginate itself has no renal-targeting properties. However, due to the presence of open-function M and G groups, it can react with other cationic polymers, such as chitosan, thus achieving renal targeting ability and the encapsulation of drugs [132].

Based on the above, alginate nanoparticles have specific applications and great potential for renal drug delivery. T. I. M. Ragab et al. [29] used alginate-encapsulated carvacrol to form nanoemulsions in order to attenuate cisplatin-induced nephrotoxicity in rats. The prepared nanoemulsions were predominantly spherical, varying in particle size between 14 and 30 nm, and negatively charged. The nanoemulsions succeeded in significantly alleviating oxidative stress and inflammation in the kidneys of model rats, showing good renoprotective properties. In a study conducted by S. Heidarisasan et al. [133], the authors utilized alginate and chitosan together to load insulin for the treatment of diabetic nephropathy in rats. The nanoparticles had a particle size of 533 nm, a zeta potential of 20 mv, and a loading capacity of 48.83%. After oral administration of the nanoparticles, this formulation successfully reduced the blood glucose and advanced glycosylation end-product levels in rats with diabetic nephropathy and improved oxidative stress conditions in the kidneys. Shanguo Zhang et al. [134] used a W/O microfluidic emulsion template method to prepare highly spherical calcium alginate microspheres in order to encapsulate a histone deacetylase inhibitor for AKI therapy. The shape of the nanoparticles they prepared was highly spherical. The in vivo results showed that the calcium alginate microspheres loaded with histone deacetylase inhibitors were effective in attenuating the inflammatory response and macrophage infiltration of the kidney.

## 5. Conclusions

With the rapid development of nanomedicine technology, an increasing number of nanomaterials have been applied for clinical treatment. Biopolymers can be extracted from plants, animals, and other living organisms. They not only have the advantages of environmental protection and recyclability but also have excellent biocompatibility, bioavailability, and responsiveness. The purpose of renal targeted drug delivery is to increase the local effective concentration and therapeutic efficacy of the drug and to reduce the toxic side effects caused by the drug in the non-therapeutic domain. By targeting specific receptors or cells in the kidney (e.g., megalin receptors and proximal tubular epithelial cells), modifying the nanoparticles (e.g., kidney-targeting peptides, etc.), and then adjusting their particle size, surface charge, and shape, the targeted delivery of nanomedicines to the kidneys can be realized. The development of nano-biopolymer systems for kidney-targeted drug delivery is a very promising area of research. Ordinary nanoparticles have the disadvantages of a low synthetic yield, high cost, poor in vivo stability, low targeting efficiency, and potential toxicity to non-target organs, and biopolymer-based nanoparticles could be a good solution to these problems. The research techniques for the synthesis of various biopolymers and their modification methods are relatively mature, and these formulations have been used as carriers for nano-drug delivery in various cancers. However, there is still a gap in the application of renal drug delivery, and many of the widely studied biopolymer carriers have not been applied to renal diseases. Biopolymer-based nanosystems for kidney-targeted drug delivery have yet to be perfected. Firstly, it is necessary to select suitable carriers from a large number of biopolymers and modify them in such a way as to ensure good bioavailability, non-toxicity, and physicochemical properties. Secondly, most of the current targeting strategies for the kidney are aimed at the renal tubules, and the targeting strategies for glomerular diseases are very limited. Hence, more new renal-targeting strategies need to be investigated. With the in-depth study of renal disease mechanisms and the application of nanotechnology, biopolymer-based nanosystems will play an increasingly important role in the targeting of renal diseases.

## Figures and Tables

**Table 1:** Nanomaterials for targeted drug delivery in the kidney.

Types of Nanoparticles	Subclasses	Advantages	Drawbacks	Ref.
Inorganic nanoparticles	Fe_3_O_4_ magnetic nanoparticles	Superparamagnetic, anti-inflammatory, and antioxidative stress effects.	Toxicity, complex preparation process.	[20,21]
	Gold nanoparticles	Easy to fabricate, highly stable surface chemistry, and multi-functionality.	Toxicity	[1]
	Quantum dots	Excellent photo-stability, high quantum yield, mainly used for renal photoacoustic imaging.	In vivo toxicity, distribution, metabolism, and excretion issues	[22,23]
Lipid nanoparticles	Liposome	Hydrophilic and lipophilic, surface modifiable with targeted ligands, biocompatible.	Costly and quick to remove.	[24,25]
	Solid lipid nanoparticles (SLNs)	Biocompatible and biodegradable, high surface area.	Low drug loading, excretion of drugs under storage conditions.	[26,27]
	Nanostructured lipid carriers (NLCs)	Compared to SLNs, they have a better drug encapsulation rate, higher drug loading capacity, and lower drug spillage during storage.	Stability and storage issues.	[27,28]
	Nanoemulsion	High bioavailability, good stability, and long shelf life.	Toxicity	[29,30]
Carbon-based nanoparticles	Carbon nanotubes(CNTs)	Excellent adsorption capacity and high surface area.	Poor solubility, low biodegradability, and toxicity issues.	[31,32]
	Graphene	Excellent optical properties, electrical conductivity, and high mechanical strength.	Toxicity and cell viability issues.	[33,34]
Polymer nanoparticles	Micelles	Stable, customizable drug release characteristics on demand.	Some polymers may be limited by the immune response	[35,36]
	Synthetic polymers	Easy chemical coupling, good drug loading or encapsulation, increased drug cycle time, uniform particle size distribution, and modifiable physicochemical properties.	Limited process technology and immunogenicity.	[1,37]
	Biopolymers	Biodegradable, eliminated from the body via normal metabolic pathways, non-toxic, biocompatible, and poorly immunogenic		[1]
	Dendrimers	Surface attachable specific targeting ligands, high permeability, and enhanced solubility.	Toxicity and untimely release of drugs.	[38]
Biomimetic nanoparticles	Cell-membrane coated nanoparticles	Extended blood circulation, high biocompatibility, and low side effects.	Some cell membranes have the potential to promote tumor growth or disease progression in their own right.	[39]
	Natural protein-based nanoparticles	Biocompatibility, biodegradability, and easy size control.	High cost and rapid degradation.	[40,41]

## Data Availability

Not applicable.
